# On-Orbit Geometric Calibration from the Relative Motion of Stars for Geostationary Cameras

**DOI:** 10.3390/s21196668

**Published:** 2021-10-07

**Authors:** Linyi Jiang, Xiaoyan Li, Liyuan Li, Lin Yang, Lan Yang, Zhuoyue Hu, Fansheng Chen

**Affiliations:** 1 Key Laboratory of Intelligent Infrared Perception, Chinese Academy of Sciences, Shanghai 200083, China; jianglinyi@mail.sitp.ac.cn (L.J.); lixiaoyan@ucas.ac.cn (X.L.); liliyuan@mail.sitp.ac.cn (L.L.); yanglin@mail.sitp.ac.cn (L.Y.); yanglan3134@163.com (L.Y.); huzhuoyue@mail.sitp.ac.cn (Z.H.); 2 CAS Key Laboratory of Infrared System Detection and Imaging Technology, Shanghai Institute of Technical Physics, Shanghai 200083, China; 3 University of Chinese Academy of Sciences, Beijing 100049, China; 4 Hangzhou Institute for Advanced Study, University of Chinese Academy of Sciences, Hangzhou 310024, China

**Keywords:** remote sensing, imaging sensor, geometric calibration, relative motion, stellar trajectory

## Abstract

Affected by the vibrations and thermal shocks during launch and the orbit penetration process, the geometric positioning model of the remote sensing cameras measured on the ground will generate a displacement, affecting the geometric accuracy of imagery and requiring recalibration. Conventional methods adopt the ground control points (GCPs) or stars as references for on-orbit geometric calibration. However, inescapable cloud coverage and discontented extraction algorithms make it extremely difficult to collect sufficient high-precision GCPs for modifying the misalignment of the camera, especially for geostationary satellites. Additionally, the number of the observed stars is very likely to be inadequate for calibrating the relative installations of the camera. In terms of the problems above, we propose a novel on-orbit geometric calibration method using the relative motion of stars for geostationary cameras. First, a geometric calibration model is constructed based on the optical system structure. Then, we analyze the relative motion transformation of the observed stars. The stellar trajectory and the auxiliary ephemeris are used to obtain the corresponding object vector for correcting the associated calibration parameters iteratively. Experimental results evaluated on the data of a geostationary experiment satellite demonstrate that the positioning errors corrected by this proposed method can be within ±2.35 pixels. This approach is able to effectively calibrate the camera and improve the positioning accuracy, which avoids the influence of cloud cover and overcomes the great dependence on the number of the observed stars.

## 1. Introduction

At present, geostationary remote sensing cameras (RSCs) are widely used in earth observation and space surveillance, such as the monitoring of ocean change, meteorological and natural disasters [1,2,3]. Geostationary RSCs can continuously monitor and quickly revisit any location within the field of regard of the satellite, which offers new functionalities not covered by low earth orbit satellites [4]. Precise orientation parameters of the camera are preset in ground-based laboratories before launch, including the principal point, the focal length and camera installation matrix with respect to the satellite-body coordinate system, which contributes to establish a geometric positioning model for direct georeferencing [5,6,7]. Nevertheless, owing to the launch vibration and the variation of spatial thermal environment, the positioning model will inevitably change, which will ultimately bring about the reduction of the geometric accuracy [8,9]. Therefore, on-orbit geometric calibration with the references including the ground control points (GCPs), coastlines and stars [10,11,12,13,14] is an essential prerequisite for ensuring the high-precision satellite imagery [15,16,17,18]. However, for geostationary RSCs, subjected to the cloud coverage and the accuracy of corresponding extraction algorithms, it is not easy to ensure the availability of a great number of accurate GCPs through earth observation, and, additionally, the star-based calibration method is usually limited by the number of the observed stars. Hence, an efficient geometric calibration method being free from the weather and the number of stars is urgently needed.

Up to now, a large number of studies have been conducted on on-orbit calibration to improve the positioning accuracy of RSCs. The early published work commonly focused on constructing the positioning models through GCPs in calibration test sites [19]. especially, according to 33 GPS-surveyed GCPs in the Denver test site, the calibration of in-flight field angle map was performed to reduce the interior orientation systematic errors of the camera of IKONOS, and the residual errors were within ±1 pixel [20,21,22]. Based on the globally distributed test sites, Valorge et al. [23] estimated the line-of-sight (LOS) biases of the Haute Resolution Géométrique carried by SPOT-5 satellite and modified the misalignment between the instruments and the attitude and orbit control subsystem (AOCS) reference frame. Similarly, using a viewing geometry model, given ephemeris and attitude data, precise camera geometry and datum transformation, Radhadevi et al. [24] address the in-flight calibration, consisting of alignment calibration of individual sensors and calibration between the sensors, for IRS-P6, which requires as many GCPs as possible for stability and reliability. Furthermore, according to the calibration fields located in Denver and Lunar Lake, the planimetric accuracy of GeoEye can be achieved as 3 m (RMS) [25,26,27]. However, relying heavily on the high-precision references of the calibration sites and the number of the manually selected GCPs, the methods above usually turn out to be inefficient and unstable.

To reduce the dependence on calibration sites and improve the efficiency, automatic GCPs extraction methods based on geographic references, including the digital orthophoto map (DOM) and the digital elevation model (DEM), as well as the associated feature matching technology, have been proposed to acquire abundant GCPs. Using the correlation method [28] to match the Orbita hyperspectral satellite images and the corresponding DOM images in Hubei area automatically, Jiang et al. [29] carried out the geometric calibration of Zhuhai-1 with 2102 extracted GCPs, and the calibration accuracy could be better than 0.5 pixels. For implementing the automatic geometric calibration of GF4, Wang et al. [30] adopted the SIFT algorithm to extract the GCPs from the DOM and DEM references of Landsat 8 and ASTER GDEM (GDEM2), and the matching accuracy was declared to be better than 0.3 pixels. Using the GPU-ASIFT automatic GCPs extraction algorithm, Dong et al. [31] obtained thousands of GCPs from the Landsat8 images and AW3D30 DSM to perform the on-orbit geometric calibration for GF-4, and the final calibration accuracy could be within 1.19 pixels. For correcting geo-referencing errors of KMSS-2 images of Meteor-M No. 2–2 satellite, Zhukov et al. [32] performed geometric calibration based on the bank of Landsat GCPs. In addition, in most cases, the errors after corrected did not exceed 60 m. Combined rational polynomial coefficient (RPC) model-based forward and inverse transformation with the DEM data extraction, Ye et al. [33] designed and implemented the automatic orthorectification system (GF1AMORS), and the experiments showed that the automatic orthorectification process exhibited a nice accuracy and stability in both mountainous terrain and plain terrain. Seo et al. [34] presented the direct geo-referencing model of KOMPSAT-3A AEISS-A using GCP/Image Control Point (ICP) to correct the distortion with under 0.5-pixel accuracy and bundle adjustment, and then the image data were provided to users. Coupled with the Gaofen-7 satellite data, Liu et al. [35] constructed a geometric imaging model of the area array footprint camera and proposed a coarse-to-fine “LPM-SIFT + Phase correlation” matching strategy for the automatic extraction of calibration control points. Compared with the calibration result using a small number of manually collected control points, the root mean square error (RMSE) of the residual of the control points is improved from half a pixel to 1/3, and the RMSE of the same orbit checkpoints in the image space is improved from 1 pixel to 0.7. Li et al. [14] proposed an accurate geometric texture-based GCPs extraction approach for the thermal infrared remote sensing images of Landsat 8 and GLS 2000, and the absolute matching errors in sample and line directions could be 0.50 and 0.47 pixels. In addition, similarly, the coastlines could also be considered as efficient references to accomplish the on-orbit calibration as well [36]. According to the GCPs obtained from the global self-consistent hierarchical high-resolution shoreline and the coastline template matching, Chen et al. [37] developed an on-orbit installation matrix calibration approach for the navigation of the advanced geostationary radiation imager (AGRI) on FY-4A with the navigation error being 1.3 pixels. Although the GCP extraction approaches above are conductive to the automatic calibration, it is obvious that they depend heavily on the cloud coverage of the images and the distribution of GCPs.

However, due to the unpredictable cloud coverage and certain features changing greatly compared with the reference image, eligible remote sensing images cannot always be obtained in real time, which results in great difficulties for the conventional methods in performing immediate calibration for urgent positioning requirements.

To avoid the GCP restrictions, Delvit et al. [38] proposed an auto-reverse method for geometric calibration of Pleiades-HR using a couple of images from the same orbit with inverse directions. Although this method works well without external references, it is not applicable to other satellites without the extreme agility.

Additionally, being independent of GCPs, star-based geometric calibration, unaffected by the eclipse and interfering daylight, is also a promising and effective method [39]. Kim et al. [40] proposed a geometric calibration using stellar sources in an earth observation satellite, which can help monitor the geographic location accuracy of satellite images. In addition, numerical simulation verified the effectiveness of the method. Using the ensemble of star field images, Christian et al. [41] proposed a geometric calibration of the Orion optical navigation camera and verified the effectiveness of the method through numerical experiments. In addition, with the stars as the reference points, Fourest et al. managed to perform the geometric calibration of Pleiades-HR [42]. Likewise, Li et al. [4] constructed a rigorous stellar-based geometric positioning model for geostationary cameras and proposed a thermal deformation positioning error correction method with the accuracy of less than ±1.9 pixels. In addition, processing the star map from the camera and star sensors for the star coordinate acquisition, Guan et al. [43] developed a camera-star sensor installation calibration method for Luojia 1-01 Satellite, which achieved a positioning accuracy of better than 800 m. Although the star-based method has some advantages over conventional methods with GCPs, it is impracticable in cases where only a few or even a single star appears in the camera’s field of view, because inadequate stars will result in a lack of references for estimating the calibration parameters.

As mentioned above, to avoid the restrictions of GCPs and the observed stars, in this paper, we propose a novel on-orbit geometric calibration method using the relative motion of observed stars for geostationary cameras. Thanks to the relative motion between the observed stars and the camera, the stellar trajectories from consecutive multi-frames are used to calculate the abundant object vectors (OVs) for correcting the calibration parameters iteratively, which, effectively, overcomes the number limitation of the observed stars. Section 2 elaborates the preprocessing of the stellar trajectory, the proposed geometric calibration method from the relative motion, and the solution of the method. Section 3 presents the experiments and results with on-orbit observation data. Section 4 focuses on discussing the findings of the study. Finally, the conclusions are summarized in Section 5.

## 2. Methodology

### 2.1. Preprocessing of Stellar Trajectory

In terms of the observation stars, the prediction of stellar trajectories can be performed according to stellar constellations and satellite attitudes. Continuous observations of stars are completed by a two-dimensional pointing mirror. We obtain a series of the star images by controlling the optical axis of the camera and making the stars move from the left to the right in the field of view (FoV).

Firstly, for each star image, the centroid of the star needs to be determined accurately. As shown in Equation (1), the centroids of the star images, generally distributed in multiple pixels, are acquired through the widely used traditional centroid extraction method [44]. The gray value of the pixel is considered to be the weight of the corresponding position for computing the center of the target.

(1) $$\begin{array}{l} x_{0}=\frac{\sum_{x\in W}\sum_{y\in W}x\times G(x,y)}{\sum_{x\in W}\sum_{y\in W}G(x,y)}\\ y_{0}=\frac{\sum_{x\in W}\sum_{y\in W}y\times G(x,y)}{\sum_{x\in W}\sum_{y\in W}G(x,y)} \end{array}$$

where *W* is the size of target window, G(x,y) is the gray value of pixel in (x,y), and $\left(x_{0},y_{0}\right)$ is the centroid position of the target.

Subsequently, with the relative motion of stars, the star trajectory can be obtained from the multiple consecutive images. Theoretically, the trajectory should be a smooth curve. However, affected by the disturbance of satellite platform, the instability of the pointing mirror, and error in discrete sampling, the actual trajectory generally presents as a series of irregular scattered points [45], which results in a centroid position error and affects the subsequent positioning accuracy. Therefore, in order to improve the accuracy of the star’s position, a smoothing spline is adopted to fitting the trajectory of the scattered star imaging points. The model can be described as

(2) $$S\left(g\right)=p\sum_{i}w_{i}{\left[y_{i}-g\left(x_{i}\right)\right]}^{2}+\left(1-p\right)\int \left(\frac{d^{2}g}{dx^{2}}\right)dx,$$

where *p* is the smoothing parameter defined in [0,1], $w_{i}$ is the weight of each point, and *g* is the function of smoothing spline fitting chosen to minimize the value of Equation (2).

### 2.2. Geometric Calibration Model

**Figure 1:**
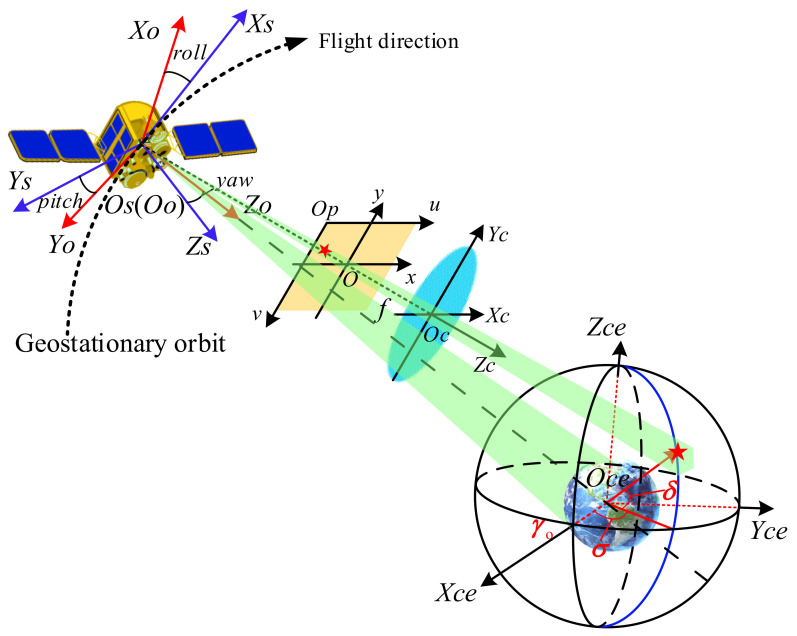
Diagram of the geometric positioning model of the geostationary RSC. *Op-u-v*, *O-x-y*, *Oc-XcYcZc*, *Os-XsYsZs*, *Oo-XoYoZo* and *Oce-XceYceZce* are the pixel coordinate system, the image coordinate system, the camera coordinate system, the satellite body coordinate system, the orbital coordinate system, and the celestial coordinate system, respectively. x-axis and y-axis are parallel to u-axis and v-axis respectively. *Xc*-axis and *Yc*-axis are parallel to x-axis and y-axis, respectively. *Os* and *Oo* is located in the centroid of the satellite. *Xs* points in the flight direction of the satellite, Ys is along the horizontal axis of the satellite, and *Zs* is determined by the right-hand rule. *Xo* points in the direction of satellite motion, *Zo* points to the center of the earth, and *Yo* is determined according to the right-hand rule. *Xce* points the vernal equinox $\gamma_{0}$, *Yce* is perpendicular to *Xce* in the equatorial plane, and *Zce* is perpendicular to the equatorial plane and points to the celestial pole.

The geometric positioning model of the camera establishes the relationship between the image point in the focal plane and the corresponding object in the geodetic coordinate system. Figure 1 shows the diagram of the geometric positioning model of the geostationary camera. During operation, the satellite continuously adjusts its attitude to make the camera face the earth for observation. Due to the high altitude and the large observable range of the geostationary camera, the star observation could be realized by controlling the camera to point to the deep space with a two-dimensional pointing mirror.

**Figure 2:**
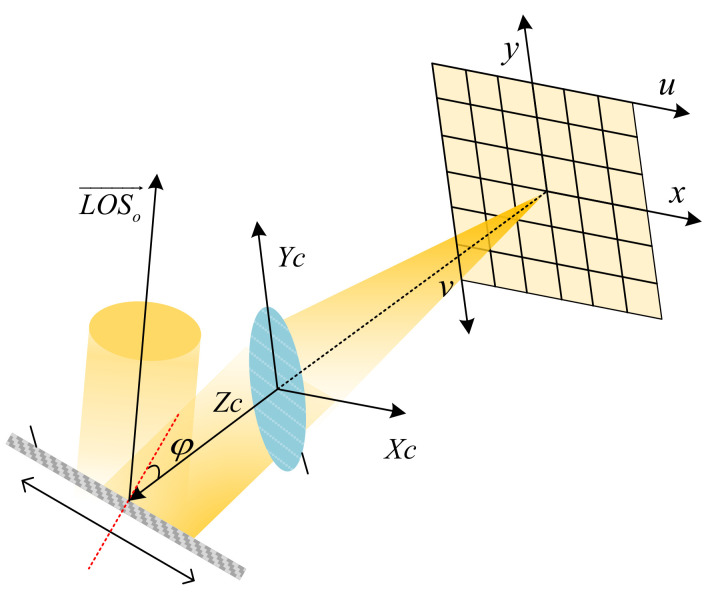
Schematic of the imaging system of camera. N is the normal vector, and φ is the intersection angle of the optical axis and the normal of the pointing mirror. is the exit vector corresponding to the principle point of the camera.

The rigorous geometric positioning model of the camera can be constructed as

(3) $$\left[\begin{array}{c} \cos\delta \cos\sigma \\ \cos\delta \sin\sigma \\ \sin\delta \end{array}\right]=\lambda R_{o-ce}R_{s-o}\left(pitch,roll,yaw\right)R_{c-s}\left(\alpha ,\beta ,\gamma \right)R_{ref}\left(\varphi \right)\left[\begin{array}{ccc} dx & 0 & \Delta x\\ 0 & dy & \Delta y\\ 0 & 0 & -f \end{array}\right]\left[\begin{array}{c} u-u_{0}\\ v_{0}-v\\ 1 \end{array}\right],$$

where (u,v) is the pixel coordinate of the image point, $\left(u_{0},v_{0}\right)$ is the pixel coordinate of the principal point *O*, Δx and Δy are the distortions of the image point in the x and y directions on the image plane coordinate system respectively, dx and dy are the dimensions of a pixel in the x and y directions, respectively, and f is the focal length of the optical system. As shown in Figure 2, $R_{ref}\left(\varphi \right)$ denotes the reflection matrix of the pointing mirror, φ is the intersection angle of the optical axis and the normal of the pointing mirror, α, β, and γ are the three installation angles of the camera mounted on the satellite relative to the three axes of the satellite body coordinate system, $R_{c-s}(\alpha ,\beta ,\gamma )$ is the corresponding installation matrix, pitch, roll and yaw are the pitch, roll and yaw angles of the satellite body coordinate system relative to the orbital coordinate system, $R_{s-o}(pitch,roll,yaw)$ is the corresponding the transformation, $R_{o-ce}$ is the transformation matrix from the orbital coordinate system to the celestial coordinate system, and λ is the scale factor.

In Equation (3), the transformation from the pixel coordinate system to the camera coordinate system and the transformation from the camera coordinate system to the celestial coordinate system can be described as the interior positioning model and exterior positioning model, respectively. Practically, the interior positioning model is often affected by the errors including the detector translation, the lens distortion, and the principal distance deviation, while the exterior positioning model is affected by the orbit measurement error, the attitude measurement error, and the camera installation error. Therefore, it is necessary to consider the influences of various error sources so as to construct a suitable calibration model.

In view of the complex interior error sources and the strong coupling between the interior parameters [6,31], a third-order polynomial is adopted to model the tangent of directional angles of the detector to avoid excessive over-parameterization [30], and the interior calibration model can be expressed as

(4) $$\left[\begin{array}{c} \tan\left(\psi_{x}\left(u,v\right)\right)\\ \tan\left(\psi_{y}\left(u,v\right)\right)\\ -1 \end{array}\right]=\left[\begin{array}{c} x_{c}/f\\ y_{c}/f\\ -1 \end{array}\right]=\frac{1}{f}\left[\begin{array}{c} x_{c}\\ y_{c}\\ -f \end{array}\right]=\frac{1}{f}\left[\begin{array}{ccc} dx & 0 & \Delta x\\ 0 & dy & \Delta y\\ 0 & 0 & -f \end{array}\right]\left[\begin{array}{c} u-u_{0}\\ v_{0}-v\\ 1 \end{array}\right],$$

(5) $$\left\{\begin{array}{c} \tan\left(\psi_{x}\left(u,v\right)\right)\\ \tan\left(\psi_{y}\left(u,v\right)\right) \end{array}\right.=\left\{\begin{array}{c} a_{0}+a_{1}u+a_{2}v+a_{3}uv+a_{4}u^{2}+a_{5}v^{2}+a_{6}u^{2}v+a_{7}uv^{2}+a_{8}u^{3}+a_{9}v^{3}\\ b_{0}+b_{1}u+b_{2}v+b_{3}uv+b_{4}u^{2}+b_{5}v^{2}+b_{6}u^{2}v+b_{7}uv^{2}+b_{8}u^{3}+b_{9}v^{3} \end{array}\right.,$$

where $\psi_{x}(u,v),\psi_{y}(u,v)$ are the directional angles of the image point (u,v), and $a_{0},\cdots ,a_{9}$, $b_{0},\cdots ,b_{9}$ are the interior parameters.

Then, to reduce the computational complexity, a generalized installation matrix $R_{ins}$ of the camera is introduced as

(6) $$R_{ins}=R_{s-c}\left(\alpha ,\beta ,\gamma \right)R_{ref}=\left[\begin{array}{ccc} A_{1} & A_{2} & A_{3}\\ B_{1} & B_{2} & B_{3}\\ C_{1} & C_{2} & C_{3} \end{array}\right],$$

where α,β,γ are the exterior parameters, $A_{1},A_{2},A_{3},B_{1},B_{2},B_{3},C_{1},C_{2},C_{3}$ are the elements in the generalized installation matrix $R_{ins}$.

Subsequently, based on Equations (3)–(6), the geometric calibration model is constructed as

(7) $$\left[\begin{array}{c} \cos\delta \cos\sigma \\ \cos\delta \sin\sigma \\ \sin\delta \end{array}\right]=\lambda R_{o-ce}R_{s-o}\left(pitch,roll,yaw\right)R_{ins}\left[\begin{array}{c} \tan\left(\psi_{x_{c}}\left(u,v\right)\right)\\ \tan\left(\psi_{y_{c}}\left(u,v\right)\right)\\ -1 \end{array}\right],$$

In the calibration model, both $R_{o-ce}$ and $R_{s-o}$ can be calculated from the attitude and ephemeris of the satellite. The exterior parameters $X_{E}$ are used to describe the synthesis of the reflect matrix and installation matrix and to compensate the installation angle error and measurement error. Similarly, the interior parameters $X_{I}$ are used to describe and compensate for the internal distortion of the camera. Using the stellar track points, the exterior and interior parameters could be computed iteratively. Distinctly, the accuracy of exterior and interior parameters determines the accuracy of the calibration model.

### 2.3. Model Solution Method

#### 2.3.1. Relative Motion Transformation

To determine the calibration parameters, sufficient references generally referring to the geographic data are usually required for the model calculation. In this paper, the celestial coordinate of the star is used to solve the model parameters. Given the star’s coordinates and the ephemeris, the OVs are introduced to represent the incident vector of the star in the satellite body coordinate system as

(8) $$OV=\left[\begin{array}{c} T_{x}\\ T_{y}\\ T_{z} \end{array}\right]=R_{o-s}R_{ce-o}P_{s},$$

where $P_{s}={\left[\cos\delta \cos\sigma \;\cos\delta \sin\sigma \;\sin\delta \right]}^{T}$ is the orientation vector of the star in the celestial coordinate system.

In the conventional star-based calibration methods [39,42], according to Equation (7), the OVs of the stars in a single frame can be expressed as $OV_{i}=R_{o-s}R_{ce-o}P_{s_{i}}(i=1,2,\cdots ,n)$ (n means the number of the stars), where $P_{si}$ is the orientation vector of the ith observed star in the celestial coordinate system. In addition, plenty of OVs are the crucial inputs to ensure that the positioning model can be solved iteratively. However, if there are only a few stars, or even single star in one frame, the obtained OVs are insufficient to support the subsequent calculation.

**Figure 3:**
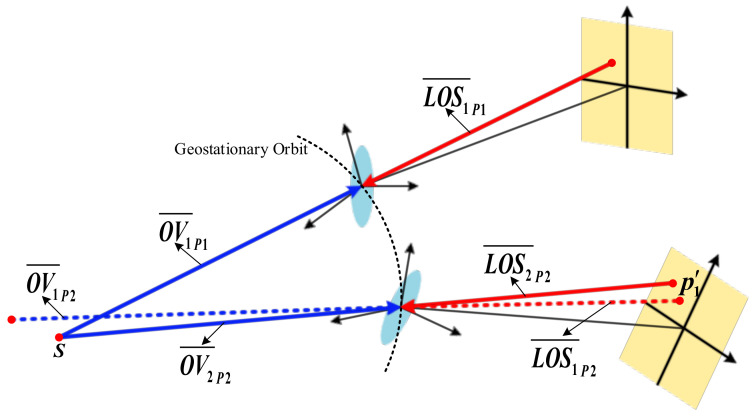
The schematic diagram of relative transformation.

To address this problem, we propose a method to obtain multiple OVs through the relative motion of the stars. As shown in Figure 3, at the position *Pos*_1_, *p*_1_ on the focal plane *FP*_1_ is the corresponding image point of the star *S*. In the satellite body coordinate system $O_{s_{1}}-X_{s_{1}}Y_{s_{1}}Z_{s_{1}}$, ${\bar{LOS_{1}}}_{P1}$ is the emergent LOS of *p*_1_, and ${\bar{OV_{1}}}_{P1}$ is the OV of *S*. Then, after reaching the position *Pos*_2_, on the focal plane *FP*_2_, *p*_2_ is the corresponding image point of the star *S*, and *p*′_1_ is at the same position as *p*_1_ on the focal plane *FP*_1_. In the satellite body coordinate system $O_{s_{2}}-X_{s_{2}}Y_{s_{2}}Z_{s_{2}}$, ${\bar{LOS_{2}}}_{P2}$ is the emergent LOS of *p*_2_, ${\bar{LOS_{1}}}_{P2}$ is the emergent LOS of *p*′_1_, and ${\bar{OV_{2}}}_{P2}$ is the OV of *S*. During operation, $O_{2}-x_{2}y_{2}$ and $O_{s_{2}}-X_{s_{2}}Y_{s_{2}}Z_{s_{2}}$ are the corresponding coordinate system of $O_{1}-x_{1}y_{1}$ and $O_{s_{1}}-X_{s_{1}}Y_{s_{1}}Z_{s_{1}}$, respectively. According to the geometric relationship of imaging, the OV coincides with the corresponding LOS. Therefore, we can obtain

(9) $$\left\{\begin{array}{c} \left\|{\bar{OV_{1}}}_{P1}\right\|=-\left\|{\bar{LOS_{1}}}_{P1}\right\|\\ \left\|{\bar{OV_{2}}}_{P2}\right\|=-\left\|{\bar{LOS_{2}}}_{P2}\right\| \end{array}\right.,$$

Since the interior and exterior parameters of the imaging system could be regarded as invariant during the operation, the emergent LOS of the image points at the same position on the focal plane are unchanged, namely, ${\bar{LOS_{1}}}_{P1}={\bar{LOS_{1}}}_{P2}$. Assume that there is a virtual star *S′* in the object space, making ${\bar{OV_{1}}}_{P2}={\bar{OV_{1}}}_{P1}$, where ${\bar{OV_{1}}}_{P2}$ is the OV of *S*′ in $O_{s_{2}}-X_{s_{2}}Y_{s_{2}}Z_{s_{2}}$. Based on the above relationship expressions, it is easy to prove that $\left\|{\bar{OV_{1}}}_{P2}\right\|=-\left\|{\bar{LOS_{1}}}_{P2}\right\|$, which denotes that *p*′_1_ could be regarded as the corresponding image point of *S*′ according to the imaging geometry principle. In other words, the stellar track points *p*_2_ and *p*′_1_ can be considered as the image of the two different stars *S* and *S*′ taken at the same time.

Then, we need to figure out the values of the OVs of *p*′_1_ and *p*_2_. In Equation (8), it can be proved that $R_{ce-o}$ is determined by the instantaneous position vector $\vec{P}(t)$ and velocity vector $\vec{V}(t)$ of the satellite, and $R_{o-s}$ also varies all the time owing to the change of the three attitude angles. Thus, the OVs of *p*′_1_ and *p*_2_ can be obtained with the different $R_{ce-o}$ and $R_{o-s}$ calculated in the position *Pos*_1_ and *Pos*_2_, respectively.

**Figure 4:**
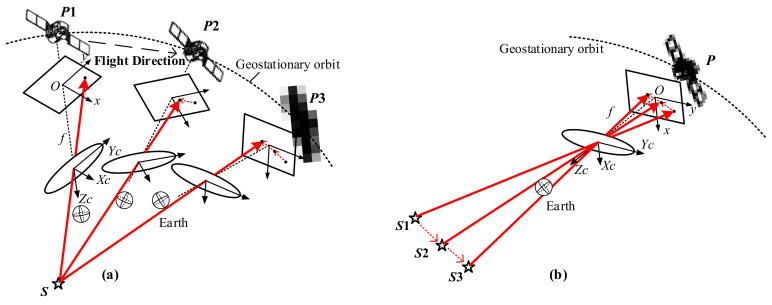
Schematic diagram of geometric transformation of trajectory. (**a**) The stellar trajectory obtained by snapping the star S at the position $P_{1},P_{2},P$. (**b**) The image of the stars $S_{1},S_{2},S_{3}$ taken at the position P.

On the basis of the theory above, multiple OVs could be obtained through the relative motion of the star. As shown in Figure 4, during the operation, the position of the star relative to the satellite changes all the time. In a short period, the interior and exterior parameters of the camera could be considered to be invariant. Since the camera geometry remains unchanged, the stellar trajectory generated by the images taken at different times can be regarded as the image of the multiple stars observed at the same time. Therefore, using enough stellar track points, we construct the OVs as $OV_{i}=R_{o-s_{i}}R_{ce-o_{i}}P_{s}(i=1,2,\cdots ,m)$ (define m as the number of the points).

#### 2.3.2. Model Solving

According to the different calculation order of the calibration parameters, the geometric calibration methods are mainly divided into three types: overall calibration, first exterior calibration and then interior calibration, and first interior calibration and then exterior calibration. To reduce the correlation between the parameters and improve the accuracy of the interior calibration parameters, this paper adopts a stepwise calibration method [30] to calculate the calibration parameters. First, the exterior parameters are estimated. Then, the interior parameters are estimated in the reference camera frame determined by the estimated exterior parameters.

According to the mentioned above equations, we transform Equation (7) as

(10) $$\left[\begin{array}{c} \tan\left(\psi_{x_{c}}\left(u,v\right)\right)\\ \tan\left(\psi_{y_{c}}\left(u,v\right)\right)\\ -1 \end{array}\right]=\lambda \left[\begin{array}{ccc} A_{1} & B_{1} & C_{1}\\ A_{2} & B_{2} & C_{2}\\ A_{3} & B_{3} & C_{3} \end{array}\right]\left[\begin{array}{c} T_{x}\\ T_{y}\\ T_{z} \end{array}\right],$$

Then, Equation (11) can be transformed from Equation (10) for the calibration.

(11) $$\left\{\begin{array}{c} S=-\frac{A_{1}T_{x}+B_{1}T_{y}+C_{1}T_{z}}{A_{3}T_{x}+B_{3}T_{y}+C_{3}T_{z}}-\tan\left(\psi_{x_{c}}\left(u,v\right)\right)\\ L=-\frac{A_{2}T_{x}+B_{2}T_{y}+C_{2}T_{z}}{A_{3}T_{x}+B_{3}T_{y}+C_{3}T_{z}}-\tan\left(\psi_{y_{c}}\left(u,v\right)\right) \end{array}\right.,$$

where S and L are the residual expressions in the horizontal direction and vertical direction, respectively.

In terms of exterior calibration, we initialize the interior and exterior parameters with the on-ground calibration parameters, and then, for each stellar track point, linearize Equation (11) to construct the error equation Equation (12) as

(12) $$V_{E}=A\Delta X_{E}-L_{E},\;P_{E}=E,$$

where A is the coefficient matrix of Equation (12), $\Delta X_{E}$ is the correction of exterior parameters, $L_{E}$ is the error vector calculated by the current interior and exterior parameters, and $P_{E}$ is the identity weight matrix. For each stellar track point, $\Delta X_{E}$ can be estimated by the least-square method as

(13) $$\Delta X_{E}={\left(A^{T}P_{E}A\right)}^{-1}\left(A^{T}P_{E}L_{E}\right),$$

Then, exterior parameters $X_{E}$ could be updated as

(14) $$X_{E}=X_{E}+\Delta X_{E},$$

$X_{E}$ is updated iteratively until $\Delta X_{E}\leq \varepsilon$, where ε is the preset small positive threshold for exterior calibration.

For interior calibration, insert the modified $X_{E}$ above into Equation (11), and, for each stellar track point, linearize Equation (11) to construct the error equation, Equation (15), as

(15) $$V_{I}=B\Delta X_{I}-L_{I},\;P_{I}=E,$$

where B is the coefficient matrix of Equation (15), $\Delta X_{I}$ is the correction of the interior parameters, $L_{I}$ is the error vector calculated by the current interior and exterior parameters, and $P_{I}$ is the identity weight matrix. Then, we can obtain the correction of interior parameters by the least-square method as

(16) $$\Delta X_{I}={\left(B^{T}P_{I}B\right)}^{-1}\left(B^{T}P_{I}L_{I}\right),$$

Interior parameters $X_{I}$ could be updated as

(17) $$X_{I}=X_{I}+\Delta X_{I},$$

$X_{I}$ is updated iteratively until $\Delta X_{I}\leq \xi$, where ξ is the preset small positive threshold for interior calibration.

#### 2.3.3. Representation of Error

On the basis of the geometric calibration model and its solution above, we have obtained the calibration parameters $X_{E}$ and $X_{I}$. To evaluate the calibration accuracy, the calibration model with the calculated calibration parameters inserted is adopted to compute the celestial coordinate of the star corresponding to the image point, and then compared with the theoretical coordinate derived from the star catalog.

The absolute positioning errors of the right ascension and declination directions are respectively defined as

(18) $$\begin{array}{l} RA_{error}=\sigma^{\prime }-\sigma \\ DE_{error}=\delta^{\prime }-\delta \end{array}$$

where $\sigma^{\prime }$ and $\delta^{\prime }$ are the practical right ascension and declination of the star computed by the calibration model, σ and δ are the right ascension and declination of the corresponding star determined from the wide-field infrared survey explorer catalog according to the observation plan, and $RA_{error}$,$DE_{error}$ are the absolute positioning errors in the right ascension and declination directions respectively. According to Equation (18), the unit of RAerror and Deerror is degree or arcsecond, while the unit of positioning error is usually pixel or meter. To express the positioning error more intuitively, based on the angle of view and the size of the detector, transform the units of Raerror and Deerror from degree to pixel as:

(19) $$Error_{\left(pixel\right)}=Error_{\left(\deg\right)}/\left(A/S\right),$$

where $Error_{\left(\deg\right)}$ and $Error_{\left(pixel\right)}$ are the positioning errors in degree and pixel respectively, *A* is the angle of view of a single detector’s response element to the optical system, and *S* is the size of the detector, which, as used in the experiment in this paper, is 1024 pixels.

## 3. Experiment and Results

According to the theories above, we evaluated the proposed method based on the real star observation short-wave infrared data of the staring camera of a geostationary experiment satellite. The detailed information of the experiment satellite is shown in Table 1. We collected the observations from August 2 to August 21 as the data sets, and the specific experimental results are shown in the following.

### 3.1. Trajectory Fitting Results

Considering that there are many stellar trajectories, a stellar trajectory of the star FYID1070664 observed on 2nd August is taken as an example to illustrate the experimental results of curve fitting.

**Figure 5:**
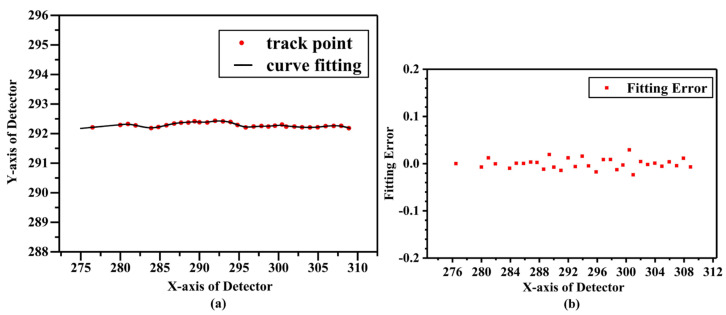
Curve fitting of stellar trajectory. (**a**) The fitting results of smoothing spline method. (**b**) The distribution of the fitting errors.

The fitting results and the distribution of fitting errors are shown in Figure 5a,b, respectively. As shown in Figure 5a, the fitting result is a smooth curve, and most of the track points are centered on the curve. It can be seen from Figure 5b that the fitting errors are generally within ±0.03 pixels. Moreover, Table 2 reveals that the sum of squares error (SSE) is close to 0 and the determination coefficient of R-Square is close to 1, which illustrates the effectiveness of the fitting model. Thus, it could be seen that the deviation of the collected stellar trajectory from the ideal trajectory points caused by the satellite platform jitter, the pointing error of the pointing mirror and the sampling error could be reduced by the curve fitting. The fitting curve could more accurately describe the positions of the stellar track points, thereby improving the subsequent calibration accuracy.

### 3.2. Results of Positioning Errors

In the process of calibration, the more track points there are, the more accurate the estimated calibration parameters will be. To improve the performance of the proposed method, stellar trajectories with more than 25 track points were selected for the experiments. For each trajectory, five track points were randomly selected as test data for the positioning errors, and the remaining points were used to calculate the parameters.

Due to the great fluctuation in the thermal environment around the geostationary satellite, the space thermal deformation resulting from it will significantly change the installation angles of the remote sensing camera and affect the positioning accuracy ultimately. Li et al. [4] found that the positioning error caused by the space thermal deformation is periodic, with a cycle of one day approximately. Therefore, to ensure the stability of the experiment, the data collected at a particular time (from 11:25 to 11:45 in this paper) on 20 consecutive days were picked for the experiments.

According to the calibration model, we calculate the positioning errors determined by the initial parameters calibrated on the laboratory and the calibration parameters through the proposed method, respectively. As shown in Table 3, compared to the initial right ascension positioning accuracy of −6.795 pixels and the initial declination positioning accuracy of −21.004 pixels, the geometric positioning errors after calibration are greatly reduced. It can be seen that the positioning accuracy after correction is approximately ±0.85 pixels, andthus we need to experiment with more data to verify the positioning accuracy of the calibration model subsequently.

**Figure 6:**
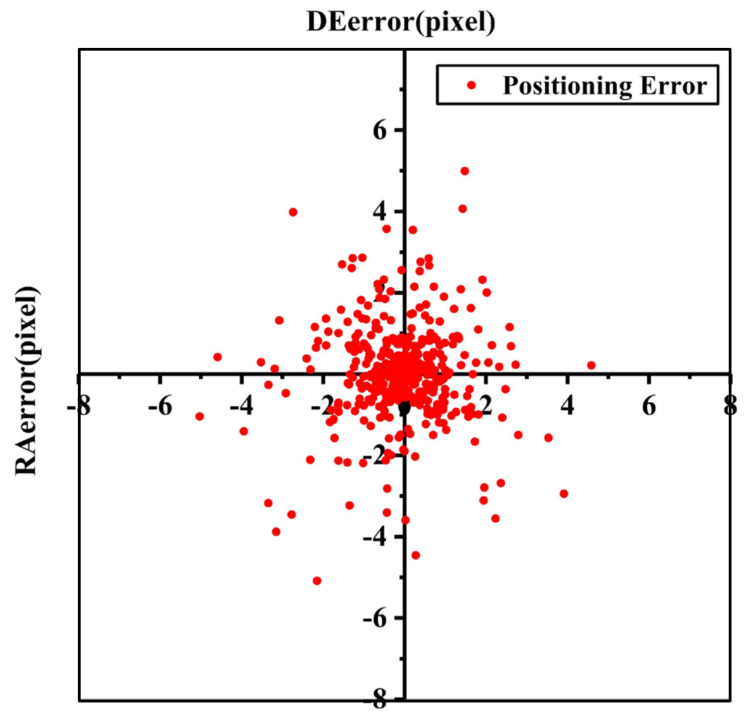
Distribution of the positioning error.

**Figure 7:**
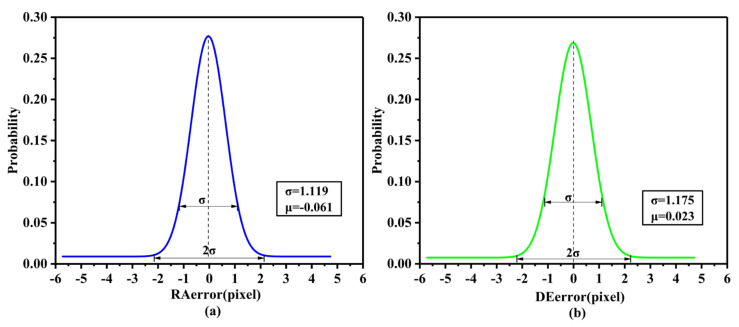
(**a**) Probability distribution of $RA_{error}$. (**b**) Probability distribution of $DE_{error}$.

The 437 stellar trajectories of the whole data set for the 20 days were used to verify the accuracy of the model. As shown in Figure 6, the positioning errors are concentrated in the middle and scattered around, which seems to accord with the normal distribution. To explore the property of the positioning errors, then we analyze the probability distribution of the positioning errors and try to fit it with the normal distribution function. The results are shown in Figure 7 and Table 4. It can be seen that the positioning errors of both the right ascension and declination directions all stably obey normal distribution. The probability of $RA_{error}$ within ±2.24 pixels at a 95% confidence level can reach 95.44% (2σ). Similarly, the probability of $DE_{error}$ within ±2.35 pixels at a 95% confidence level can reach 95.44% (2σ). According to the experimental results of the real data of on-orbit satellite, it demonstrates that the proposed approach corrects the periodic positioning errors caused by the space thermal deformation.

## 4. Discussion

By analyzing the experimental results of the data collected at a specific time on 20 consecutive days, the absolute positioning errors after calibration were reduced from 21.004 pixels to 0.85 pixels. Subsequently, 437 stellar trajectories were used to verify the accuracy of the method. In addition, the results show that the positioning errors in the right ascension and declination directions corrected by this proposed method obey normal distribution stably and can be within ±2.35 pixels. In addition, the distribution of positioning errors also explains why the minimum absolute error in Table 3 is about 0.1 pixel, and the maximum is about 3.0 pixels. Due to the random selection of the test track points, that is, the track points for calibration were selected randomly, the estimation of the parameters with different points are different, which affects the positioning error. Most absolute positioning errors falls within 2.35 pixels, and occasionally the absolute positioning errors are about 3 pixels.

**Figure 8:**
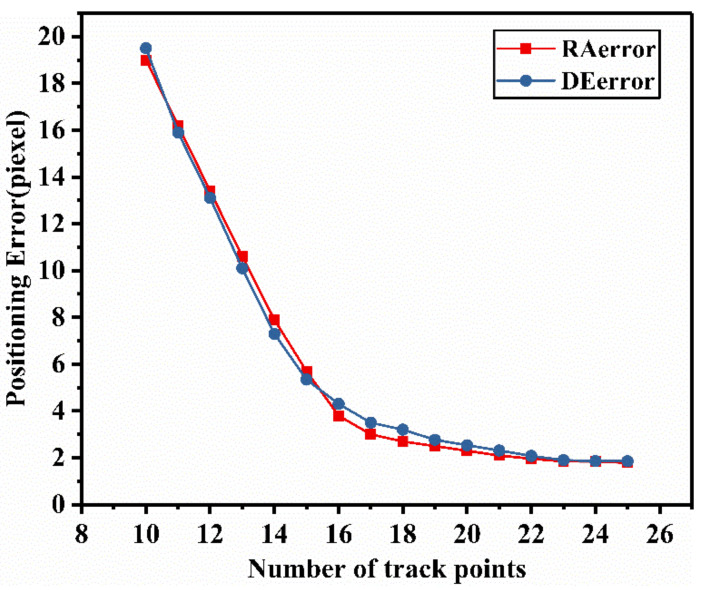
Influence analysis for the number of track points on the positioning accuracy.

Furthermore, to further analyze the influence of the number of track points on the calibration accuracy, the experiments with 10 to 25 track points for calibrating have been carried out and the results were shown below. As shown in Figure 8, the positioning errors kept reducing with the increase of the number of track points. When more than 20 calibrating track points are available, the reduction of positioning error gradually slows down and the error becomes stable. This is because the least squares method is used to estimate the parameters. When there are fewer track points, the accuracy of the algorithm is greatly dependent on the number of samples. Increasing the number of the track points will obviously improve the accuracy of parameter estimation, thereby improving the calibration accuracy. When the number reaches a certain degree, the accuracy of the algorithm tends to be stable. Therefore, the reduction in the number of track points will affect the positioning accuracy.

It should be noted that, taking into account the characteristics of the normal distribution, the positioning accuracy of the model is also affected by other random errors. Random measurement errors such as orbit measurement error and attitude measurement error may be one of the key factors that determine the calibration accuracy of this method. In addition, the accuracy of star centroid extraction and trajectory fitting accuracy also directly affect the positioning accuracy of the model, which may also be a further study in the future.

## 5. Conclusions

For a geostationary remote sensing camera, due to the orbital heat flux and the shock and vibration during launch, the installation structures between the camera and satellite platform will change inevitably and ultimately bring about the reduction of the positioning accuracy, which needs recalibrating. Nevertheless, traditional on-orbit camera calibration methods with GCPs and stars are often affected by cloud coverage or limited to the number of stars. To address these problems, this paper presents a novel on-orbit geometric calibration method from the relative motion of stars for geostationary cameras. Based on the optical system structure, a geometric calibration model is constructed. In addition, then the relative motion transformation of the observed stars is analyzed. According to the analysis above, we adopt the stellar trajectory and the auxiliary ephemeris to get sufficient input OVs for estimating the calibration parameters iteratively.

The proposed method is verified with on-orbit measurement data. Experimental results demonstrate that the positioning model can be well-calibrated by the proposed approach and the geometric accuracy of the remote sensing images is significantly improved. With the increase of the number of track points, the calibration accuracy is gradually improved. Though, this method is proposed for geostationary cameras, it is likely to be suitable and versatile for other RSCs because of the similar spatial relative motion relationship between the satellite and the target stars.

## Figures and Tables

**Table 1:** Parameters of the experiment satellite.

Items	Detailed Parameters
Orbit altitude	36,000 km
Focal length	1250 mm (short-wave infrared)
Array sensor information	1024 × 1024 HgCdTe
Pixel size	25 μm (short-wave infrared)
Accuracy of attitude measurements	$1\times 10^{-4}{}^{\circ}/s$

**Table 2:** Analysis of fitting accuracy.

	SSE	R-Square	RMSE
**Orbit altitude**	0.003856	0.9776	0.01728

SSE is the sum of squares error, R-Square is the determination coefficient of the model and RMSE is the root-mean-squared error. The closer the SSE approaches 0, the R-Square approaches 1, the better the fitting effectiveness becomes.

**Table 3:** Positioning errors before and after calibration.

	Initial Positioning Errors		Positioning Errors after Calibration			
	RAerror/Pixel	DEerror/Pixel	RAerror/Pixel	Absolute Error/Pixel	DEerror/Pixel	Absolute Error/Pixel
2nd August	−10.384	−20.247	−0.616	0.616	−0.080	0.08
3rd August	−5.081	−20.430	−0.086	0.086	0.099	0.099
4th August	−6.402	−20.508	−0.484	0.484	2.892	2.892
5th August	−5.181	−20.624	−0.464	0.464	1.233	1.233
6th August	−5.336	−20.563	−1.210	1.21	−0.233	0.233
7th August	−5.877	−20.557	−0.017	0.017	−0.127	0.127
8th August	−7.975	−20.208	−0.110	0.11	−0.008	0.008
9th August	−4.686	−20.414	−0.063	0.063	0.027	0.027
10th August	−5.713	−21.664	1.642	1.642	−0.736	0.736
11th August	−8.704	−20.375	−0.568	0.568	0.468	0.468
12th August	−4.839	−21.312	−0.100	0.1	−0.446	0.446
13th August	−5.371	−21.283	−0.271	0.271	−0.437	0.437
14th August	−8.423	−20.874	−1.571	1.571	0.025	0.025
15th August	−8.366	−21.446	−2.038	2.038	0.984	0.984
16th August	−5.023	−22.626	1.076	1.076	2.793	2.793
17th August	−7.060	−21.679	−0.280	0.28	−1.177	1.177
18th August	−8.879	−20.206	3.712	3.712	1.505	1.505
19th August	−7.201	−22.269	0.511	0.511	1.013	1.013
20th August	−7.743	−21.209	−0.888	0.888	1.864	1.864
21st August	−7.658	−22.182	−1.128	1.128	0.921	0.921
Mean	−6.795	−21.004	−0.148	0.84175	0.529	0.8534

**Table 4:** Analysis of the probability distribution for the positioning error.

	CL	mu	muci	sigma	sigmaci
**RAerror**	95%	−0.0611	(−0.1661, 0.0440)	1.1187	(1.0492, 1.1982)
**DEerror**		0.0234	(−0.0870, 0.1338)	1.1754	(1.1024, 1.2589)

CL is the confidence level. mu and sigma are the estimate values of the mean and the standard deviation, respectively. muci and sigmaci are the confidence interval of mu and sigma, respectively.

## Data Availability

Data sharing not applicable.
